# Case of Carotid Aneurism

**Published:** 1830

**Authors:** 


2. *Case of Carotid Aneurism where the Artery was taken up above the Tumour.*—In the last number of this journal we took notice of the successful performance of this operation by Dr.

Mott. The Medico Chirurgical Review contains an account of another successful operation by A. Montgomery, Surgeon to the Civil Govnt. Hosp. at Mauritius.

The subject of this very formidable disease was a free black, aged about 40 years, a creole of this colony, and was employed as a gent d'armes ; tall, of a square habit of body, and rather given to intemperance. Was admitted into the Civil Government Hospital (of which I am surgeon in charge) on the 20th February of the present year, for an aneurismal tumour the size of a pullet's egg, situated immediately above the sternal portion of the left clavicle, and so close to that bone, that it seemed to emerge from behind it, or rather from within the cavity of the chest ; which rendered the taking up of the common carotid artery below the aneurism absolutely impracticable.

The poor man had an almost constant tickling cough, with severe pain of the trachea ; copious frothy mucous expectoration ; great anxiety of countenance ; hoarseness of vioce ; disturbed sleep ; and was rather emaciated from constant watching.

From this period the tumour went on increasingly rapid until 9th March, when it had acquired an alarming size, the base occupying the space of two-thirds of the sternal portion of the clavicle, and ascending nearly four inches upwards to the angle of the jaw, so that the volume of the tumour limited exceedingly the space for taking up the artery above it. An incision was made of an inch and a quarter long through the integments and platysma myoides muscle, by which the inner edge of the sterno cleido mastoideus was brought into view, greatly thrown inward and forward out of its natural position. This being drawn aside by a retractor, the incision was continued on its inner side in the direction of the carotid artery with every possible caution to avoid the superficial veins, one of which, of considerable size, crossed the neck, limiting very much my space for operating. The after part of the operation was attempted with a silver knife, as directed by Mr. Wardrop, but finding that instrument too clumsy, and depending on the steadiness of my hand, I removed the cellular substance, and exposed the sheath of the vessels, by dissecting with the forceps and scalpel, occasionally using the handle of the latter, when I found it necessary. This dissection exposed the descendens noni running on the front of the sheath of the vessels, the bifurcation of the artery, the external jugular vein at the upper angle of the incision, and a vein the size of a large crow quill crossing the artery at the lower angle, and immediately above the omohyoaideus muscle, which limited the place for taking up the artery to little more than half an inch. The sheath of the vessel was now slit opên, when the artery, vein, and par vagum nerve, were seen in their natural situations.

The patient who had been very restless during the whole of the operation, suddenly raised himself up so that I was compellèd to seek again for the sheath, and being unable to find the first opening, I was obliged to make a second one. An attempt was now made to pass a rude crooked aneurisnal needle armed with a double ligature around the artery, in which I was foiled by the restless state of the patient. A second attempt proved more successful, as it passed with facility. On the ligature being laid hold of, the needle was withdrawn and on one ligature being secured the other was removed and the wound brought in contact by a simple suture and strap of adhesive plaster. The patient being now faint a little wine and water was given and he was put to bed. It is remarkable that scarcely a drop of blood was lost during the operation (which was performed in about 25 minutes) except what escaped from the vessels, of the integuments and platysma myoides, which did not exceed a tea-spoonful.

By the 14th of March the tumour was diminished to half its size the pulsation only to be observed when the patient lay in a recumbent posture. And the condition of the patient in other respects much improved. 18th. The tumour still smaller but a pulsation perceptible at a small point on the humeral edge of the aneurism indicative of approaching rupture of the sac. 20th, Slight hemorrhage from the wound, which, except where the legature comes out, is entirely healed ; pulsation at the point mentioned on the 18th more distinct. The hemorrhage recurred on the 22d several times in the course of the day, but was on every occasion easily commanded.

April 7th, The tumour painful. From this time it gradually enlarged and threatened to suppurate. And, on May 29th, it gave way and discharged about 8 oz. of fetid chocolate colored fluid. No hemorrhage having taken place, it was enlarged on the next day, and about 6 or 8 oz. more of the same fluid discharged. The artery could now be felt at the bottom of the wound, without pulsation. June 8th, The patient begins to walk out, the wound is nearly healed, and the tumour has entirely disappeared.

